# Mixing Compost and Biochar Can Enhance the Chemical and Biological Recovery of Soils Contaminated by Potentially Toxic Elements

**DOI:** 10.3390/plants13020284

**Published:** 2024-01-18

**Authors:** Matteo Garau, Maria Vittoria Pinna, Maria Nieddu, Paola Castaldi, Giovanni Garau

**Affiliations:** 1 Dipartimento di Agraria, University of Sassari, Viale Italia 39, 07100 Sassari, Italy; mgarau@uniss.it (M.G.); mavi@uniss.it (M.V.P.); marianieddu@outlook.it (M.N.); castaldi@uniss.it (P.C.); 2 Nucleo Ricerca Desertificazione, University of Sassari, 07100 Sassari, Italy

**Keywords:** soil amendment, labile Sb and Zn, soil microorganisms, enzymatic activities, plant growth, Sb and Zn uptake

## Abstract

Biochar and compost are able to influence the mobility of potentially toxic elements (PTEs) in soil. As such, they can be useful in restoring the functionality of contaminated soils, albeit their effectiveness can vary substantially depending on the chemical and/or the (micro)biological endpoint that is targeted. To better explore the potential of the two amendments in the restoration of PTE-contaminated soils, biochar, compost (separately added at 3% *w*/*w*), and their mixtures (1:1, 3:1, and 1:3 biochar-to-compost ratios) were added to contaminated soil (i.e., 2362 mg kg^−1^ of Sb and 2801 mg kg^−1^ of Zn). Compost and its mixtures promoted an increase in soil fertility (e.g., total N; extractable P; and exchangeable K, Ca, and Mg), which was not found in the soil treated with biochar alone. All the tested amendments substantially reduced labile Zn in soil, while biochar alone was the most effective in reducing labile Sb in the treated soils (−11% vs. control), followed by compost (−4%) and biochar–compost mixtures (−8%). Compost (especially alone) increased soil biochemical activities (e.g., dehydrogenase, urease, and β-glucosidase), as well as soil respiration and the potential catabolic activity of soil microbial communities, while biochar alone (probably due to its high adsorptive capacity towards nutrients) mostly exhibited an inhibitory effect, which was partially mitigated in soils treated with both amendments. Overall, the biochar–compost combinations had a synergistic effect on both amendments, i.e., reducing PTE mobility and restoring soil biological functionality at the same time. This finding was supported by plant growth trials which showed increased Sb and Zn mineralomass values for rigid ryegrass (*Lolium rigidum* Gaud.) grown on biochar–compost mixtures, suggesting a potential use of rigid ryegrass in the compost–biochar-assisted phytoremediation of PTE-contaminated soils.

## 1. Introduction

Potentially toxic elements (PTEs) are natural constituents of the Earth’s crust, although human activities such as mining, industrial production, and agricultural practices can significantly increase their concentrations in soil up to toxic levels [1,2,3]. Mining activities can release PTEs into the environment due to a lack of secured abandoned mine sites, leading to widespread contamination. Therefore, effective management practices that prioritize sustainability and environmental protection, as well as public health, are required to reduce the negative environmental effects associated with disused mining sites [4,5].

To mitigate the impact of PTE contamination in soil, several approaches have been proposed, including phytoremediation, bioremediation, and chemical immobilization. The latter strategy aims to reduce the mobility and bioavailability of PTEs, thus promoting soil functionality and plant growth [5]. The application of organic amendments, such as compost and biochar, is a promising technique used for PTE immobilization which is gaining attention due to its eco-friendly and cost-effective benefits [6,7,8]. Compost is a nutrient-rich organic amendment derived from the aerobic decomposition of waste biomass, such as agri-food and lignocellulosic pruning residues under controlled conditions, while biochar is a stable carbon-rich material derived from slow pyrolysis under the limited oxygen conditions of different biomasses, e.g., wood chips, agricultural residues, and others [9]. Both organic amendments could have multiple beneficial effects on soil health, such as improved soil structure, increased water retention, and reduced soil erosion [10,11]. However, due to their peculiar physico-chemical characteristics, these amendments could influence PTE mobility and soil properties in different, but not always optimal, ways.

Compost is rich in functional groups (e.g., carboxylic, phenolic, thiophenolic, and sulfhydryl groups) that can specifically adsorb PTEs, reducing their mobility and bioavailability [2,5,12,13]. Compost can increase the soil buffering capacity, preventing the release of adsorbed PTEs into the environment as a result of pH changes [5]; compost usually contains high concentrations of bioavailable nutrients and dissolved organic carbon (DOC) which can stimulate microbial activities and plant growth, enhancing the fertility and functionality of contaminated soils [14,15]. However, there are some worrying results on the effect of compost addition to PTE-contaminated soils in the literature. For example, DOC released from compost, containing low-molecular-weight organic acids, can increase PTE mobility through the formation of soluble PTE–DOC complexes (e.g., [16,17]). Moreover, the addition of high rates (>20%) of compost to PTE-contaminated soils can have a detrimental effect on plant growth due to the excessive supply of nutrients in mobile form [18].

As mentioned above, biochar is an alternative material used for the recovery of degraded and PTE-contaminated soils due to its large surface area and porosity, as well as the presence of functional groups (e.g., hydroxyl, ester, carboxyl, and carbonyl) that allow PTE immobilization through chemical reactions such as ion exchange, cation π bonding, surface complexation, precipitation, and diffusion into pores [19,20,21,22,23,24]. Moreover, biochar alkalinity can increase soil pH, thus reducing the availability of PTEs in cationic form whose solubility is negatively correlated with soil pH [25]. An added value of biochar lies in its stability and long-lasting action that can be prolonged for several years after application [25]. At the same time, there are some drawbacks to its use. For example, biochar has a medium-to-high cost and can contribute to greenhouse gas emissions if it is not produced sustainably [26,27]. Biochar application at high doses could have harmful effects on micro- and mesofauna in soil because of the toxic substances it may contain, such as ammonium ions or polycyclic aromatic hydrocarbons [22,28]. Finally, due to its high adsorption capacities, biochar can reduce the concentration of DOC and available nutrients in soil (e.g., N, K, and P), limiting plant and microbial growth [19,29,30].

Combining compost and biochar in appropriate ratios can compensate for the described limitations of both amendments and produce synergistic effects that increase the restoration effectiveness of contaminated soils. However, as far as we know, there are very few studies reporting on the combined action of biochar and compost in PTE-contaminated soils (e.g., [3,16,17,18]). 

Accordingly, further research is needed to optimize the combined application of compost and biochar in PTE-contaminated environments and provide suitable protocols for the recovery of PTE-contaminated soils. In this context, the present study evaluated the effectiveness of biochar, compost, and a combination of the two in different ratios in restoring PTE-contaminated soil using a comprehensive approach. The effects of these amendments on the chemical, biochemical, and microbiological properties of soil; the mobility and phytotoxicity of PTEs (i.e., Sb and Zn) against rigid ryegrass (*Lolium rigidum* Gaud.); and plant growth and PTE uptake were considered. 

## 2. Results and Discussion

### 2.1. Influence of Biochar, Compost, and Their Mixtures on Soil Properties

The main chemical features of the treated and untreated soils are reported in Table 1. Biochar alone and its mixtures were the most effective at raising soil pH (approximately by 0.17 and 0.29 units) compared to U-soil; this was due to the strongly alkaline nature of this amendment (Table S1) [3,18,22,31]. Biochar treatment alone reduced the EC by 1.03-fold and the DOC content by 1.5-fold, while an opposite effect was observed in mixtures and soil treated with compost (Table 1). In all the amended soils, TOC increased between 1.08- and 1.65-fold compared to U-soil. Compost and compost/biochar mixtures increased the total N and extractable P by 1.11- and 1.27-fold, and 1.90- and 3.38-fold, respectively, compared to the untreated soil. CEC increased in the soils amended with compost, in B25/C75- and B50/C50-soils compared to U-soil, while exchangeable K and Ca decreased of about 4- and 1-fold in B-soil. The reductions of EC, DOC, and exchangeable K and Ca in biochar-treated soil were probably attributable to the ability of this amendment to retain ions and small organic molecules on its external or internal surfaces, as reported by other authors [19,22]. The total concentration of Sb decreased in the treated soils, while that of Zn was not affected by the amendment.

The influence of biochar or compost on soil fertility depended on the ratios at which they were added to the soil. Compost was generally more effective than biochar in enhancing soil fertility parameters (e.g., total N, extractable P, exchangeable K, Ca, and Mg), although compost in combination with biochar was able to counteract the high adsorption capacity of biochar (particularly with regard to DOC and exchangeable K).

### 2.2. Influence of Biochar, Compost, and Their Mixtures on PTE Mobility

**Figure 1:**
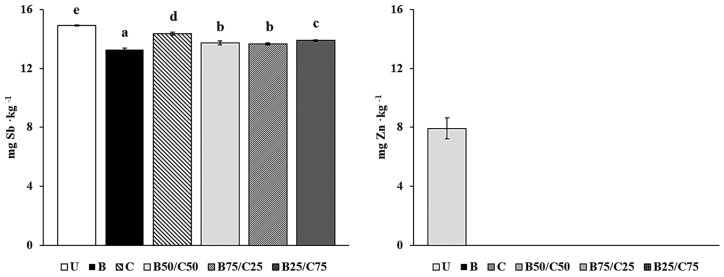
Labile Sb and Zn in the untreated soil (U) and in soils treated with biochar (B), compost (C), and biochar–compost mixtures (B50/C50; B75/C25; B25/C75). For each PTE, bars with different letters denote statistically significant differences according to the Fisher’s least significant difference (LSD) test (*p* < 0.05) (mean values ± SE; *n* = 3).

The labile fraction of Sb and Zn (i.e., their soluble and easily exchangeable pool) was determined in amended and unamended soils to evaluate the ability of biochar, compost, and their mixtures to reduce the environmental hazards associated with PTE-contaminated soils. Indeed, this fraction represents the most potentially bioavailable PTE pool, accountable for risks to the environment and human health [2,32,33]. The results showed that labile Sb was between 0.63 and 0.69% (13.3–14.9 mg∙kg^−1^) compared to its total concentration in soil, while labile Zn (in the control soil) was around 0.30% (7.9 mg∙kg^−1^). It is important to emphasize that both total and labile Sb concentrations exceeded (by ~1000- and 7-fold respectively) the Finnish threshold value (2 mg∙kg^−1^ of Sb) for contaminated soils, which represents a good approximation of the mean values of different national legislations in Europe [34]. As such, the Sb contamination in the studied soil is expected to pose serious danger to the environment and human health. The mobility of labile Sb and Zn decreased in the treated soils, in particular, Zn concentration decreased below the detection limit (<0.2 μg kg^−1^). Biochar was the most effective at reducing labile Sb, in particular, this fraction was 11% lower than that of U-soil (Figure 1). This means approx. 6 kg less labile and potentially bioavailable Sb per ha in biochar-treated soil (30 cm depth, density 1200 kg m^−3^). Compost decreased labile Sb only by 4%, while the combinations of biochar and compost, which were more effective than compost alone, decreased the mobile Sb fraction by ~8% in the order: B75/C25 = B50/C50 < B25/C75 (Figure 1). These reductions suggested the occurrence of several stable interactions between biochar (especially), compost, and their mixtures with Sb, in agreement with the results reported by other researchers [3,35]. Under normal oxidizing soil conditions, Sb is predominantly present in the form of antimonate anion (Sb(OH)_6_^−^) [36,37] and can be adsorbed by biochar and compost through different interaction mechanisms which may prevail depending on the amendment. These include: (i) the formation of stable inner-sphere complexes with Fe oxides present in biochar [22,24]; (ii) the formation of ternary complexes in which polyvalent metal cations (e.g., Ca^2+^) act as bridges between the negatively charged functional groups of compost or biochar and antimonate [2,3,24,36]; (iii) antimonate coprecipitation with Ca^2+^ (particularly abundant in compost) to form the sparingly soluble Ca(Sb(OH)_6_)_2_ [36]. The higher effectiveness of biochar to adsorb Sb(V) could be due to the higher reactivity of this amendment compared to compost and/or to its porous structure which can facilitate Sb(V) immobilization by diffusion and physico-chemical fixation.

The high effectiveness of all the treatments at reducing Zn mobility could be due to the increased pH of the amended soils, which favored the formation of insoluble compounds such as Zn(OH)_2_, and ZnCO_3_ [19,24,31,38,39]. In addition, due to the high affinity of Zn with carboxyl (–COOH) and hydroxyl (–OH) phenolic groups, biochar and compost may have retained this PTE by forming inner-sphere complexes on their surface [2,3,12,19,20,23,24,36,38].

Taken together, these results highlight that compost, biochar, and their combinations were able to reduce the Sb and Zn mobility in the amended soils. In particular, biochar alone or as a mixture was fundamental to reducing labile Sb although the high pH of the treated soils should have reduced its sorption.

### 2.3. Influence of Biochar, Compost, and Their Mixtures on Enzyme Activities and Biolog Community-Level Physiological Profile

Selected soil enzyme activities, i.e., dehydrogenase (DHG), β-glucosidase (GLU) and urease (URE), soil basal respiration, and Biolog community-level physiological profile (CLPP) were used as biological indicators to evaluate the effectiveness of biochar, compost, and their combinations at restoring the functionality of the PTE-contaminated soil.

**Figure 2:**
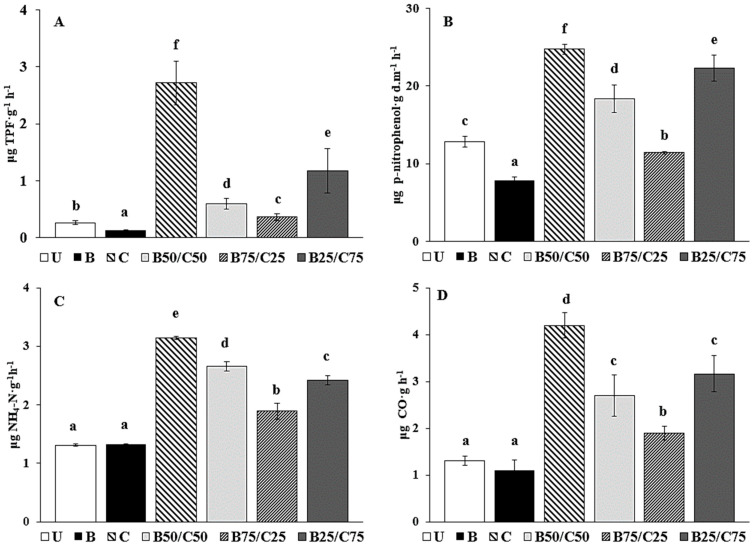
Dehydrogenase activity (DHG; (**A**)), β-glucosidase activity (GLU; (**B**)), urease activity (URE; (**C**)), and basal soil respiration (**D**) in the untreated soil (U) and in soils treated with biochar (**B**), compost (**C**), and biochar–compost mixtures (B50/C50; B75/C25; B25/C75). For each enzyme activity, bars with different letters denote statistically significant differences according to the Fisher’s least significant difference (LSD) test (*p* < 0.05) (mean values ± SE; *n* = 3).

The activity of DHG, used to estimate the oxidative capacity of soil microbial communities [40], decreased by 2.06-fold in B-soil compared to U-soil (Figure 2A). The addition of compost promoted a DHG increase of up to 10-fold compared to U-soil, and proportional to the rate of compost added, i.e., C > B25/C75 > B50/C50 > B75/C25 (Figure 2A). The effect of biochar on DHG activity of PTE-contaminated soils is controversial: while some researchers (e.g., [3,23]) observed an increase in DHG following the addition of biochar, others, such as Kaurin et al. [41] and Ali et al. [42], reported its reduction. In our study, the decrease in DHG in biochar-treated soils could be due to a reduced microbial population, which in turn could be due to a decrease in available nutrients (i.e., DOC, exchangeable K and Ca) strongly retained by the biochar [7,41,42,43,44]. On the contrary, the higher content of available nutrients in compost-treated soils (i.e., DOC, available P, total N, and exchangeable K, Ca, and Mg) might be responsible for the increased microbial number, which resulted in higher DHG as previously reported [2,14,41,45,46]. Remarkably, the significant reduction of labile Sb and Zn in the biochar-treated soil was not sufficient to enhance soil microbial activity. 

The same can be said for GLU activity, which catalyzes the hydrolysis of non-reducing terminal glucosyl residues of polysaccharides. GLU decreased by 1.62-fold in the biochar-amended soil and increased with increasing compost content (between 1.12- and 1.93-fold, Figure 2B). GLU reduction in biochar-treated soil can be attributed to the possible interaction of biochar with the enzyme, the substrate, or the enzyme–substrate complex [47,48]. At the same time, the increased content of available nutrients in the compost-treated soil (including the mixtures) may have stimulated GLU synthesis by microorganisms [2,3]. A similar trend was reported by Tang et al. [7], who observed an inhibitory effect of biochar on GLU in a PTE-contaminated soil, while compost and biochar/compost mixture increased it.

URE activity, due to extracellular enzymes capable of catalyzing the hydrolysis of urea to CO_2_ and NH_4_^+^ [49], was not affected by the addition of biochar compared to control soil, while the most effective treatment enhancing such activity was compost alone (+2.39-fold), followed by B50/C50 > B25/C75 > B75/C25 (Figure 2C). The addition of compost (alone or in mixture), by increasing soil nutrient availability, could have stimulated microbial activity resulting in increased URE synthesis, which is required to meet the nitrogen demand of the microbial community [8,50]. These results agree with those of Tang et al. [7], who reported a null influence of biochar on URE and a positive effect of compost and biochar–compost combination on such enzyme activity.

Basal soil respiration was not affected by the addition of biochar, while it increased by 2.43-fold in the C-soil compared to the control; in the mixtures, soil respiration was augmented proportionally to compost rate (Figure 2D). It is likely that the reduction of labile PTEs, combined with the new provision of mineral nutrients and easily degradable organic carbon in compost-treated soils, had a beneficial effect on the size and activity of the microbial community, as evidenced by the CO_2_ emission data. Respiration data in the different soils are consistent with those of the enzyme activities and suggested an overall reduction of the biochemical activity in the soil treated with biochar alone and a parallel increase in those treated with compost. 

**Figure 3:**
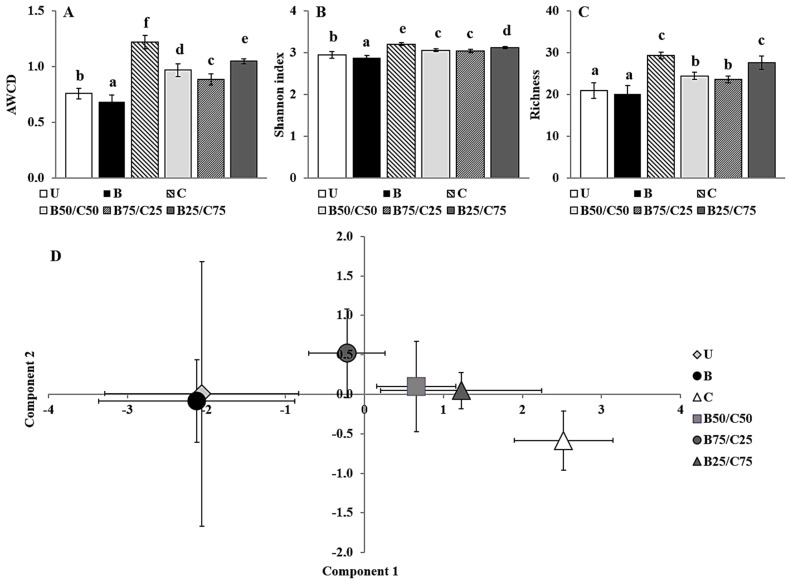
Average well color development (AWCD) (**A**), Shannon index (**B**), richness (**C**), and biplot of the PCA scores (**D**) of microbial communities extracted from the untreated soil (U) and from soils treated with biochar (**B**), compost (**C**), and biochar–compost mixtures (B50/C50; B75/C25; B25/C75). For each Biolog-derived parameter, mean values followed by different letters denote statistically significant differences according to the Fisher’s least significant difference (LSD) test (*p* < 0.05) (mean values ± SE; *n* = 3).

The Biolog CLPP is a useful tool able to highlight differences between soil microbial communities based on their respective catabolic capacities with respect to selected C sources [2,14]. The average well color development (AWCD), i.e., a measure of the overall catabolic potential of the microbial community, decreased by 1.10-fold in B-soil and increased between 1.17- and 1.60-fold in soils treated with compost alone and in combination with biochar (Figure 3A). This supported the results of the enzymatic activities, indicating that biochar can have a detrimental effect, while compost (alone or mixed with biochar) can increase them (Figure 2). Since the AWCD is commonly positively correlated with the number of culturable heterotrophic bacteria, these results also suggest that biochar may have a harmful effect on this microbial population [8,14]. In addition, biochar had a negative impact on the catabolic diversity of the microbial communities as highlighted by the Shannon index [8], which decreased by 1.02-fold in B-soil, while it increased between 1.03- and 1.08-fold in compost-treated soils compared to the control (Figure 3B). Biochar alone did not affect the number of substrates catabolized by the microbial community (richness), whereas compost (alone or mixed) increased the richness by 1.14- and 1.38-fold compared to U-soil (Figure 3C). 

Overall, the AWCD, Shannon index, and richness values indicated a significant positive impact of compost (even when mixed with biochar) on the catabolic potential and versatility of the soil microbial communities. These results, as mentioned before, could be due to an increase in the bacterial community size and related activity but also to a possible change in the community structure. To evaluate the latter possibility, C source utilization data were processed by principal component analysis (PCA; Figure 3D). PCA, which accounted for approx. 58% of the total variance in the first two components, clearly separated the different treatments along the first axis, and indicated a substantial influence of compost (which was proportional to the amount added) on the C source utilization pattern of soil microbial communities. The first principal component, which mainly separated the different treatments (47% of the total variance), was highly correlated with the consumption of selected C sources such as 4-hydroxy benzoic acid (*r* = 0.89), putrescine (*r* = 0.85), L-phenilalanine (*r* = 0.82), D-xilose (*r* = 0.80), α-cyclodextrin (*r* = 0.78), L-asparagine (*r* = 0.78), N-acetyl-D-glucosamide (*r* = 0.77), and L-serine (*r* = 0.76). Meanwhile, the second principal component (11% of the total variance) was mainly correlated with the consumption of the following substrates: glycogen (*r* = 0.46), D-cellobiose (*r* = 0.40), β-methyl-D-glucoside (*r* = 0.37), L-asparagine (*r* = 0.34), D-galacturonic acid (*r* = 0.31), and γ-hydroxybutyric acid (*r* = 0.30).

Taken together, the Biolog CLPP indicated that compost and biochar, by means of modifying soil nutrient concentration and PTE bioavailability, were able to change the soil microbial community, and this could be important for plant growth and their resilience against biotic and abiotic stresses [8,14]. 

### 2.4. Influence of Biochar, Compost, and Their Mixtures on Plant Growth

**Figure 4:**
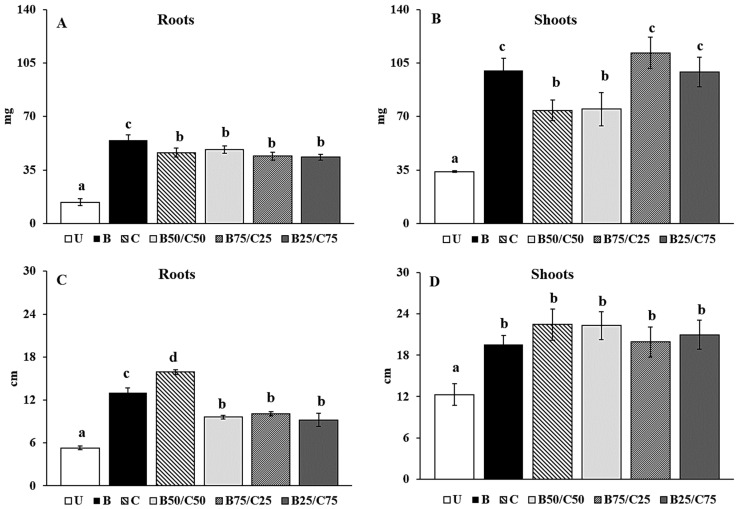
Root (**A**) and shoot (**B**) dry weight and root (**C**) and shoot (**D**) length of rigid ryegrass grown on the untreated soil (U) and on soils treated with biochar (**B**), compost (**C**), and biochar–compost mixtures (B50/C50; B75/C25; B25/C75). For each plant measure, mean values followed by different letters denote statistically significant differences (*p* < 0.05) according to the Fisher’s least significant difference (LSD) test (*p* < 0.05) (mean values ± SE; *n* = 3).

Plants can be an effective tool in environmental restoration programs as they are able to sensitively respond to environmental stress. In particular, plant growth can be a useful indicator to select effective amendments to be used in assisted phytoremediation programs. In this context, we used rigid ryegrass as a bioindicator of the remediation capabilities of compost, biochar, and their mixtures. Regardless of the amendment added, rigid ryegrass was able to grow in the untreated contaminated soil and did not develop any apparent symptom of phytotoxicity. The plant biomass detected in the U-soil was in line with that reported by Poblaciones et al. [51] for *L. rigidum* grown in a Cd-contaminated soil and by De Conti et al. [52] for *L. perenne* cultivated in a Cu-contaminated one. Importantly, biochar, compost, and their combinations enhanced plant growth. Biochar alone was the most effective at increasing the root biomass (+3.89-fold) compared to control plants (Figure 4A). Shoot biomass increased between 2.18- and 3.29-fold in plants grown in the amended soils compared to those grown in the U-soil. Biochar alone and mixtures (B75/C25 and B25/C75) were the most efficient in promoting the biomass production of rigid ryegrass shoot.

The root elongation was mostly stimulated by compost addition (+3-fold) followed by biochar alone (+2.45-fold) and biochar–compost mixtures (between 1.81- and 1.91-fold; Figure 4C). This agrees with Beesley et al. [17], who observed that aqueous extracts from a contaminated soil treated with compost, i.e., rich in available nutrients, were most effective at enhancing root elongation of *L. perenne* compared to extracts from biochar alone and biochar–compost mixture. Biochar, compost, and their combinations increased shoot elongation, but no significant differences were recorded between treatments (Figure 4D). Overall, biochar proved to be the most effective amendment in promoting biomass production, while compost favored longer root length which can be a useful trait especially in arid or drought-stressed environments.

The positive amendment effects on plant growth were likely due to the reduced PTE mobility in the treated soils (particularly evident in the B-soil), as well as to the increased nutrient content and microbial activity in soils containing compost. The combination of these two effects in the mixtures, particularly in B75/C25, might have determined a synergistic effect on the aerial biomass production of rigid ryegrass, as also reported by Karami et al. [53] for *L. perenne*, by Medyńska-Juraszek et al. [20] for green leafy vegetables, and by Hassan et al. [18] for *Arabidopsis thaliana*, grown in PTE-contaminated soils. The plant growth data also indicated that the soil microbial community of the biochar-treated soil, as well as its reduced biochemical activity, did not negatively influence the growth of rigid ryegrass. However, this does not rule out the possibility that they can have a role in plant resilience to (different) abiotic and biotic stresses.

### 2.5. Influence of Biochar, Compost, and Their Mixtures on PTE Uptake by Rigid Ryegrass

**Figure 5:**
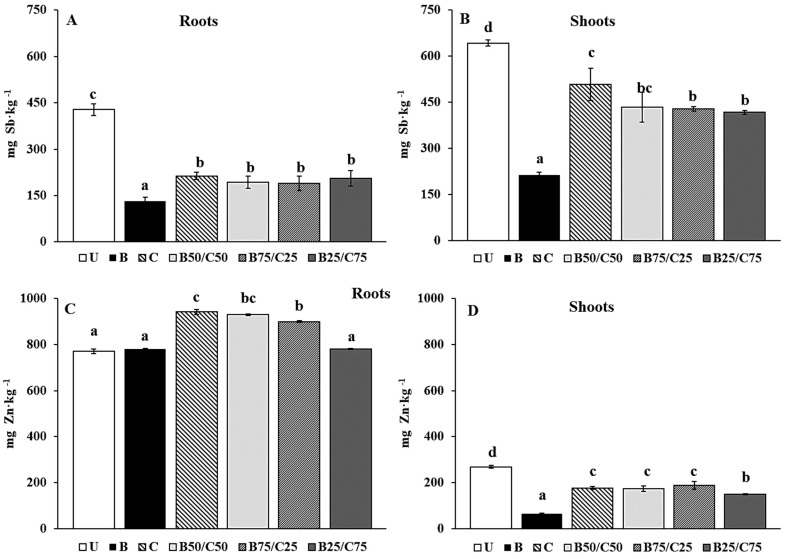
Sb (**A**,**B**) and Zn (**C**,**D**) concentration in roots and shoots of *L. rigidum* grown on the untreated soil (U) and on soils treated with biochar (**B**), compost (**C**), and biochar–compost mixtures (B50/C50; B75/C25; B25/C75). For each plant part, mean values followed by different letters denote statistically significant differences according to the Fisher’s least significant difference (LSD) test (*p* < 0.05) (mean values ± SE; *n* = 3).

Rigid ryegrass was able to take up Sb and Zn and exhibited different behaviors with respect to their accumulation in different parts of the plant. Irrespective of the treatment, Sb concentrations were higher in shoots than in roots, while the opposite was found for Zn (Figure 5). This should be due to the different bioaccumulation and detoxification mechanisms of these PTEs by *L. rigidum*. The high Sb translocation from root to shoot likely occurred to allow the conversion in the aerial part of Sb(V) (in particular) and Sb(III) into less toxic chemical species, such as methylated Sb or Sb–thiol complexes [54,55]. Rigid ryegrass accumulated very high concentrations of Zn in the roots (i.e., >700 mg kg^−1^), as also recorded for other grasses (e.g., *Arundo donax*, *Hordeum vulgare*, *L. perenne*, and *Zea mays*) grown in contaminated soils [56,57,58,59,60]. 

Biochar, compost, and their combinations reduced the Sb and Zn concentration in plant tissues (except for Zn in roots). Biochar alone was the most effective to reduce Sb in roots (−3.27-fold) and shoots (−3.01-fold), followed by biochar–compost mixtures and compost alone (Figure 5A,B). The reduced Sb uptake observed in plants grown on treated soils was probably due to the reduced mobility of this PTE in such soils. This effect was more marked for plants grown in B-soil due to the higher immobilizing capacity of biochar towards Sb compared to compost. This agrees with that reported by Hagner et al. [61], where the uptake of PTEs (i.e., As, Cr, Cu, and Ni) by *L. perenne* was lower in the presence of biochar than compost.

With regard to Zn concentration in roots, no significant differences were recorded for plants grown in B-soil and B25/C75 compared to the U-grown plants (Figure 5C). Furthermore, the addition of compost alone and selected biochar–compost mixtures (B50/C50 and B75/C25) increased Zn concentration in the roots (Figure 5C). A different trend was recorded for Zn concentration in the shoots, which decreased by 4.08-fold (compared to U-soil) for plants grown in B-soil and between 1.43- and 1.80-fold for plants grown in the other soils (Figure 5D). Despite the reduced mobility of Zn in compost-containing soils, its great uptake by the roots could be attributed to the increased availability of nutrients in these soils, which likely enhanced the metabolic activities of the roots favoring the subsequent uptake of this essential micronutrient [51,62,63].

### 2.6. Influence of Biochar, Compost, and Their Mixtures on PTE Bioaccumulation, Translocation, and Mineralomasses in Rigid Ryegrass 

In general, root and shoot BAF values, which quantify the plant’s ability to bioaccumulate PTEs in organs, were quite low, i.e., between 0.05 and 0.34 (Table 2). In all the treatments, Sb-BAF_R_ was lower than Sb-BAF_S_ but, on the contrary, Zn-BAF_R_ was higher than Zn-BAF_S_, indicating that Sb taken up from soil is preferentially transferred to the shoots, while Zn is mainly retained at the root level. The amendment addition decreased Sb-BAF, e.g., biochar alone was the most effective at reducing Sb bioaccumulation in both roots and shoots (<3-fold), while for plants grown in compost-treated soil and mixtures Sb-BAF decreased between 2.00- and 2.25-fold in roots and between 1.23- and 1.50-fold in shoots compared to control plants (Table 2). Zn-BAF_R_ was not affected by the addition of biochar and B25/C75 mixture, while the other treatments increased it in comparison to control plants. The reduction in the amended soils of BAF_R_ (except for Zn) and BAF_S_ in the rigid ryegrass confirms the effectiveness of all treatments to immobilize Sb and Zn in soil, reducing their phytoavailability and subsequent plant bioaccumulation.

The PTE translocation factor (TF), which measures the plant’s ability to transfer the PTEs from roots to shoots [64], was also determined to evaluate the possible amendment impact on this parameter. Regardless of soil treatment, Sb-TF was >1 (i.e., between 1.50 and 2.38), while Zn-TF was <1 (i.e., between 0.16 and 0.36; Table 2), confirming that rigid ryegrass actively translocated Sb from roots to shoots, while Zn was stored in root tissues. Soil amendments had an opposite effect on Sb- and Zn-TF by increasing the former and decreasing the latter. Compost alone and biochar–compost mixtures were the most effective at increasing Sb-TF and decreasing Zn-TF (except B25/C75). As stated above, although Zn uptake significantly increased in roots grown in compost-treated soils, less Zn was translocated to shoots compared to U-soil. It could be assumed that compost reduced Zn translocation by enhancing the metabolic activity of roots which favored Zn storage at the root level [65]. These findings agree with Novak et al. [66] who reported reduced Zn translocation in *Panicum virgatum* grown in compost alone and compost–biochar mixture.

PTE mineralomasses (MM_R_ and MM_S_), or the actual amounts of PTEs bioaccumulated in plant tissues, are useful to assess the plant’s ability to take up soil PTEs and potential use in phytoremediation. Irrespective of the treatment applied, Sb-MM_R_ was lower than Sb-MM_S_ (between 3.00- and 5.73-fold), while Zn-MM_R_ was higher than Zn-MM_S_ (between 1.15- and 4.05-fold). Once more, this confirmed that Sb was mainly bioaccumulated in shoots, while Zn was mostly retained in roots (Table 2). Compost alone and B50/C50 were the most effective treatments enhancing Sb- and Zn-MM_R_, respectively, while the B75/C25 mixture was the most effective at increasing Sb- and Zn-MM_S_ (Table 2). Although compost and biochar reduced the mobility of PTEs (Figure 1), the improved plant growth recorded in the amended soils increased the overall removal efficiency of Sb and Zn, albeit with important distinctions. Sb accumulation was particularly observed in the shoots of plants grown on B75/C25-soil, while Zn was mostly stored in the roots of plants grown on B50/C50-soil. Overall, biochar–compost mixtures enhanced the ability of rigid ryegrass to accumulate Sb in the aerial part and Zn in the roots. 

## 3. Materials and Methods

### 3.1. Soil Origin, Sampling, and Experimental Set-up

Soil samples (upper 30 cm) were randomly collected from a 1 ha area located in the ex-mining/smelting area of Su Suergiu (Cagliari) in southeastern Sardinia (Italy; 39°29′55″ N; 09°22′30″ E) where the main ore mined was stibnite (Sb_2_S_3_) with impurities of calcite (CaCO_3_) and quartz (SiO_2_) [67]. This site hosted the most important antimony mine in Italy for several years. The mine and the smelter for the production of antimony oxide (Sb_2_O_3_) were in operation since 1880 until the mine was closed in 1987 [67]. PTE-rich wastes were accumulated and abandoned in the vicinity of the mining plant, without taking any effective action to mitigate their environmental impact on the surrounding area [67]. The sampled soils were mixed in the laboratory, air-dried, sieved to <2 mm, and used to set up different mesocosms. Texture analysis classified the soil as sandy soil (USDA classification; i.e., 90.08% sand, 2.66% silt, and 7.26% clay).

The mesocosms, each consisting of approximately 8 kg of sieved soil (<2 mm), were treated separately by adding 3% (*w*/*w*) of a softwood-derived biochar (B), 3% of a municipal solid waste compost (C), or 3% of a mixture consisting of: 50% biochar and 50% compost (B50/C50); 75% biochar and 25% compost (B75/C25); and 25% biochar and 75% compost (B25/C75). Unamended soil was used as control (untreated soil, U). Each mesocosm was replicated three times, so 18 mesocosms in total were set up. Biochar, derived from pyrolysis at 700 °C of elder, beech, and poplar softwood, was supplied by Ronda SpA (Zanè, Italy). Compost, obtained by municipal solid waste and green waste composting, was provided by Verde Vita Srl (Sassari, Italy). The main chemical characteristics of both amendments are reported in Table S1. 

After amendment addition, treated and untreated soils were thoroughly mixed and wetted to 40% of their water-holding capacity (WHC). The mesocosms were incubated at constant humidity for 2 months at 20 °C and during this time they were mixed once a week.

### 3.2. Soil Sample Characterization and Analytical Determinations

After the incubation period, soil samples from each mesocosm were air-dried and their main physico-chemical properties were determined. Soil pH and electric conductivity (EC) were measured in 1:5 (*w*/*v*) solid-to-water suspensions (ISO 10390:2021; Table 1). Total organic carbon (TOC) and nitrogen were determined with a CHN analyzer (Leco CHN628) using Soil LCRM Leco part n° 502–697 as a calibration sample. The dissolved organic carbon (DOC) was quantified as previously described by Manzano et al. [19]. The cation exchange capacity (CEC) and exchangeable Na, K, Ca, and Mg were determined using the BaCl_2_-triethanolamine method [68], while the extractable P was determined following the Olsen method [68].

The total concentration of PTEs (i.e., Cd, Cr, Cu, Ni, Pb, Sb, and Zn) was determined by an inductively coupled plasma optical emission spectrometer (Perkin Elmer Optima 7300 DV ICP-OES) after microwave digestion (ultraWave Microwave Milestone) of soil samples with suprapure H_2_O_2_ and a HNO_3_ + HCl (3:1 *v*/*v* ratio) mixture. The NIST-SRM 2711A certified reference soil was included for quality assurance. The measured PTE values for the certified material were between 93 and 104% of the certified values.

Only the mobility of those PTEs (i.e., Sb and Zn) whose total concentration exceeded the limits set by the Italian legislation for industrial areas [69] were considered. To assess labile (i.e., water-soluble and readily exchangeable) Zn, 1 g of a soil sample was treated with 25 mL of a 0.5 M Ca(NO_3_)_2_ solution for 16 h at 20 °C [70]. Labile Sb (i.e., water-soluble and non-specifically sorbed) was determined by treating 1 g of a soil sample with 25 mL of a 0.05 M (NH_4_)_2_SO_4_ solution for 4 h at 20 °C [33]. After treatment with the extracting solutions, samples were centrifuged and filtered to separate the liquid and the solid phases. The concentration of PTEs in the filtered solutions was quantified as described above.

### 3.3. Enzyme Activities, Soil Basal Respiration, and Microbial Carbon

After the incubation period, fresh soil samples from each mesocosm were characterized biochemically. Selected enzyme activities were determined as reported by Alef and Nannipieri [71]. Briefly, the dehydrogenase activity (DHG) was quantified as triphenyltetrazolium formazan released during 24 h of incubation at 30 °C from triphenyltetrazolium chloride; the urease activity (URE) was quantified as N-NH_4_^+^ released from urea after 2 h of incubation at 37 °C; the β-glucosidase activity (GLU) was quantified as p-nitrophenol released from p-nitrophenyl glucoside in soil samples incubated for 1 h at 37 °C [71].

Soil basal respiration was determined following the procedure reported by Marabottini et al. [72], through the quantification of CO_2_ developed in soil samples (20 g) incubated at 25 °C for 7 days by NaOH titration with 0.1 M HC1. 

### 3.4. Community-Level Physiological Profile

After the incubation period, the CLPP of the microbial communities of each mesocosm was determined by means of Biolog EcoPlates (Biolog Inc., Hayward, CA, USA) as described by Garau et al. [38]. Microbial communities were extracted from soil samples with sodium pyrophosphate and inoculated into the wells of a Biolog EcoPlate while the C source utilization was determined every 24 h by recording the absorbance values in each well at 590 nm (OD590) using a Biolog MicroStation microplate reader (Biolog, Hayward, CA, USA). Normalized OD590 values (Garau et al. [38]) measured after 96 h, which provided the best discrimination between treatments, were used to calculate the Biolog-derived indexes (average well color development (AWCD), Shannon index, richness) and to carry out principal component analysis (PCA) using the correlation matrix. 

### 3.5. Plant Growth Experiment and Plant Analysis

After the soil amendment contact period, 5 pots (7 cm diameter, 6 cm height) were set up for each mesocosm, each containing 100 g of soil. A total of 90 pots were prepared (15 replicated pots × 6 treatments). Rigid ryegrass (*L. rigidum* Gaud. cv. Nurra) seeds were pre-germinated in 1 mM CaCl_2_ solution and, subsequently, three germinated seeds with ~1 cm roots were planted in each pot. This species, robust and adaptable to environmental stress, such as salinity, herbicides, and PTEs [51], was chosen as a bioindicator plant to evaluate the effectiveness of the different treatments at restoring the functionality of the contaminated soil and not to test the possibility of growing food or feed crops in such soil. The plants were grown over 3 months in a greenhouse under controlled conditions (20–25 °C air temperature and 60–70% air relative humidity). The experiment was stopped after 90 days when the plants were at the beginning of the tillering stage to keep the aerial and root parts separated. At harvest, the shoots and roots were separated, washed carefully with ultrapure water, and measured in height; the plants were then dried at 55 °C for 72 h. Plant tissues were mineralized by microwave digestion (ultraWave Microwave Milestone) with a solution containing suprapure H_2_O_2_ and a mixture of 69% HNO_3_ and ultrapure H_2_O (ratio 1:1). Subsequently, Sb and Zn concentrations in roots and shoots were determined by ICP-OES (Perkin Elmer Optima 7300 DV). Peach leaves (NIST-SRM 1547) were used as standard reference material for quality assurance. The measured values of Sb and Zn were between 95 and 106% of the certified values.

The PTE bioaccumulation and translocation factors and mineralomasses (MMs) were calculated as follows [64,73]:

- BAF_R_: ratio between the PTE concentration in roots and that present in soil;
- BAF_S_: ratio between the PTE concentration in shoots and that present in soil;
- TF: ratio between the PTE concentration in shoots and that present in roots;
- MM_R_: root biomass × PTE concentration in roots;
- MM_S_: shoot biomass × PTE concentration in shoots.

### 3.6. Data Analysis

All the analyses were performed in triplicate and mean values ± standard errors (SEs) are shown in figures and tables. One-way analysis of variance (one-way ANOVA) was used to compare mean values of the different treatments. When significant *p*-values were obtained (*p* < 0.05), the differences between the individual means were compared using Fisher’s post hoc least significance difference test (LSD, *p* < 0.05). All statistical analyses were carried out using the NCSS 2007 Data Analysis software (v. 07.1.21; Kaysville, UT, USA).

## 4. Conclusions

The results of this study showed that compost (and not biochar) added at 3% to an Sb- and Zn-contaminated soil significantly increased its chemical fertility (e.g., DOC, exchangeable K and Ca, available P, and CEC) as well as its biochemical and microbiological properties. On the other hand, biochar added at 3% was more effective than compost and biochar–compost mixtures at reducing Sb mobility, while all treatments reduced labile Zn comparably. Biochar–compost mixtures showed a synergistic action by reducing the concentration of labile Sb and Zn in soil, restoring soil biochemical functionality and microbial diversity, promoting plant growth, and reducing PTE uptake by rigid ryegrass. Soil chemistry, biochemistry, and plant growth data suggested that biochar–compost mixtures (i.e., B50/C50 and B25/C75 above all) could be an effective solution to exploit the benefits of both biochar and compost for the functional restoration of Sb- and Zn-contaminated soils. Such options appear as good candidates for a field trial aimed to confirm their effectiveness. Overall, our results showed that chemical data alone (e.g., the reduction of labile PTEs in the amended soils) are not sufficient to predict possible consequences on the soil microbial and/or biochemical status, while the latter is not indicative of plant growth potential. A more comprehensive or holistic approach considering different (micro)biological proxies and the use of different plant species as bioindicators, in addition to soil chemical data, may be more suitable in the evaluation of soil restoration options. Finally, our results indicate that the impact of softwood biochar (pyrolyzed at high temperature) on soil chemistry and functionality should be carefully (re)considered especially in terms of biochemical activity and soil microbial diversity. Further studies are also needed to establish the duration over time of the observed effects and the suitability of biochar–compost mixtures (also addressing different biochar types and/or other ratios) for the functional recovery of different PTE-contaminated soils under different field and climatic conditions. This latter aspect will be essential to assess the practicality and scalability of these amendments for real-world applications.

## Figures and Tables

**Table 1:** Chemical characteristics of the untreated soil (U) and of soils treated with biochar (B), compost (C), biochar/compost mixture 1:1 (B50/C50), biochar/compost mixture 3:1 (B75/C25) and biochar/compost mixture 1:3 (B25/C75) (mean ± standard error (SE)) *.

	U	B	C	B50/C50	B75/C25	B25/C75
pH	7.61 ± 0.04 ^a^	7.90 ± 0.03 ^e^	7.68 ± 0.02 ^b^	7.78 ± 0.01 ^c^	7.86 ± 0.01 ^d^	7.81 ± 0.02 ^c^
EC (mS cm^−1^)	2.37 ± 0.01 ^b^	2.30 ± 0.02 ^a^	2.99 ± 0.04 ^f^	2.62 ± 0.03 ^d^	2.47 ± 0.01 ^c^	2.85 ± 0.02 ^e^
Total organic matter (%)	6.43 ± 0.17 ^a^	10.64 ± 0.22 ^d^	6.93 ± 0.14 ^b^	8.77 ± 0.21 ^c^	9.50 ± 0.70 ^c^	7.24 ± 0.09 ^b^
Total N (%)	0.26 ± 0.02 ^a^	0.29 ± 0.01 ^ab^	0.33 ± 0.03 ^b^	0.30 ± 0.01 ^b^	0.29 ± 0.01 ^ab^	0.32 ± 0.03 ^b^
TOC (%)	3.73 ± 0.10 ^a^	6.17 ± 0.13 ^d^	4.02 ± 0.09 ^b^	5.09 ± 0.21 ^c^	5.51 ± 0.24 ^c^	4.20 ± 0.10 ^b^
DOC (mg·g^−1^)	0.03 ± 0.00 ^b^	0.02 ± 0.00 ^a^	0.20 ± 0.01 ^e^	0.07 ± 0.00 ^c^	0.04 ± 0.00 ^b^	0.15 ± 0.01 ^d^
Extractable P (mg·kg^−1^)	5.09 ± 0.23 ^a^	5.20 ± 0.22 ^a^	17.23 ± 0.56 ^e^	12.24 ± 0.42 ^c^	9.65 ± 0.23 ^b^	15.36 ± 0.53 ^d^
CEC (cmol_(+)_·kg^−1^)	16.05 ± 0.06 ^a^	14.86 ± 0.09 ^a^	17.69 ± 0.03 ^c^	16.98 ± 0.11 ^b^	15.93 ± 0.07 ^a^	17.38 ± 0.05 ^c^
Exchangeable Na (cmol_(+)_·kg^−1^)	0.06 ± 0.01 ^a^	0.10 ± 0.02 ^b^	0.47 ± 0.00 ^f^	0.27 ± 0.02 ^d^	0.16 ± 0.01 ^c^	0.33 ± 0.01 ^e^
Exchangeable K (cmol_(+)_·kg^−1^)	0.12 ± 0.01 ^b^	0.03 ± 0.00 ^a^	0.42 ± 0.04 ^d^	0.24 ± 0.01 ^c^	0.12 ± 0.02 ^b^	0.26 ± 0.02 ^c^
Exchangeable Ca (cmol_(+)_·kg^−1^)	14.81 ± 0.10 ^b^	14.15 ± 0.21 ^a^	16.29 ± 0.38 ^d^	15.73 ± 0.44 ^c^	14.73 ± 0.23 ^b^	16.00 ± 0.11 ^cd^
Exchangeable Mg (cmol_(+)_·kg^−1^)	0.23 ± 0.03 ^a^	0.29 ± 0.02 ^b^	0.49 ± 0.02 ^d^	0.39 ± 0.00 ^c^	0.37 ± 0.05 ^c^	0.41 ± 0.00 ^c^
						
*Total PTEs (mg·kg* *^−1^* *)*						
Cd	11.91 ± 1.33					
Cr	47.12 ± 1.99					
Cu	136.18 ± 4.74					
Ni	122.02 ± 9.56					
Pb	402.96 ± 6.34					
Sb	2362 ± 26 ^b^	2080 ± 23 ^a^	2070 ± 12 ^a^	2072 ± 19 ^a^	2068 ± 129 ^a^	2089 ± 21 ^a^
Zn	2801 ± 224 ^a^	2791 ± 51 ^a^	2801 ± 174 ^a^	2822 ± 106 ^a^	2782 ± 208 ^a^	2818 ± 83 ^a^

* Mean values followed by different letters denote statistically significant differences according to the Fisher’s least significant difference (LSD) test (*p* < 0.05).

**Table 2:** PTE bioaccumulation factors (root BAF_R_ and shoot BAF_S_; mean ± SE), translocation factor (TF; mean ± SE), and mineralomasses (root MM_R_ and shoot MM_S_; mg plant^−1^, mean ± SE) of *L. rigidum* grown on the untreated soil (U) and on soils treated with biochar (B), compost (C), and biochar–compost mixtures (B50/C50; B75/C25; B25/C75) *.

		U	B	C	B50/C50	B75/C25	B25/C75
BAF_R_	Sb	0.18±0.01 ^c^	0.06 ± 0.01 ^a^	0.09 ± 0.01 ^b^	0.08 ± 0.01 ^b^	0.08 ± 0.01 ^b^	0.09 ± 0.01 ^b^
	Zn	0.27 ± 0.00 ^a^	0.28 ± 0.00 ^a^	0.34 ± 0.00 ^c^	0.33 ± 0.01 ^bc^	0.32 ± 0.01 ^b^	0.28 ± 0.01 ^a^
BAFs	Sb	0.27 ± 0.00 ^d^	0.09 ± 0.00 ^a^	0.22 ± 0.03 ^c^	0.18 ± 0.02 ^b^	0.18 ± 0.00 ^b^	0.18 ± 0.00 ^b^
	Zn	0.10 ± 0.00 ^d^	0.06 ± 0.00 ^b^	0.06 ± 0.00 ^b^	0.05 ± 0.00 ^a^	0.06 ± 0.00 ^b^	0.07 ± 0.00 ^c^
TF	Sb	1.50 ± 0.05 ^a^	1.63 ± 0.07 ^b^	2.38 ± 0.16 ^d^	2.24 ± 0.15 ^d^	2.26 ± 0.03 ^d^	2.03 ± 0.02 ^c^
	Zn	0.36 ± 0.06 ^c^	0.21 ± 0.03 ^ab^	0.19 ± 0.04 ^a^	0.16 ± 0.03 ^a^	0.20 ± 0.03 ^a^	0.25 ± 0.02 ^b^
MM_R_	Sb	5.98 ± 0.04 ^a^	7.13 ± 0.05 ^b^	9.89 ± 0.03 ^f^	9.36 ± 0.05 ^e^	8.35 ± 0.06 ^c^	8.93 ± 0.05 ^d^
	Zn	10.77 ± 0.02 ^a^	42.50 ± 0.05 ^d^	43.68 ± 0.03 ^e^	44.92 ± 0.03 ^f^	39.67 ± 0.04 ^c^	33.88 ± 0.03 ^b^
MMs	Sb	21.81 ± 0.06 ^b^	21.41 ± 0.08 ^a^	37.61 ± 0.36 ^d^	32.52 ± 0.53 ^c^	47.82 ± 0.07 ^f^	41.39 ± 0.06 ^e^
	Zn	9.35 ± 0.03 ^a^	16.44 ± 0.03 ^d^	13.09 ± 0.06 ^c^	11.09 ± 0.13 ^b^	20.30 ± 0.04 ^f^	19.46 ± 0.03 ^e^

* Mean values followed by different letters denote statistically significant differences according to the Fisher’s least significant difference (LSD) test (*p* < 0.05).

## Data Availability

Data are contained within the article.

## Supplementary Materials

The following supporting information can be downloaded at: https://www.mdpi.com/article/10.3390/plants13020284/s1, Table S1: Chemical characteristics of the biochar and compost used in this study.
