# Supplementary material for: Mixing Compost and Biochar Can Enhance the Chemical and Biological Recovery of Soils Contaminated by Potentially Toxic Elements
Source: Plants (Basel). 2024 Jan 18;13(2):284. doi: 10.3390/plants13020284 (PMC10818981; doi:10.3390/plants13020284)
Supplement: Supplementary file 1 [file plants-13-00284-s001.zip › Supplementary material Table S1.pdf]

Table S1 Chemical characteristics of the biochar and compost used in this study. Values represent mean ± SE (n=3).
u.d.l.: under detection limit (i.e., <0.2 μg·kg^{-1}).

Chemical parameters	Biochar	Compost
pH	9.30±0.01	8.60 ± 0.02
EC (mS·cm^{-1})	9.91±2.79	4.59 ± 0.01
Ash (%)	2.44±0.10	46.42 ± 2.84
CEC (cmol$_{(+)}$ kg^{-1})	18.81±0.30	77.75 ± 0.21
Total C (%)	61.32±0.06	26.38 ± 0.39
Total N (%)	0.30±0.02	2.80 ± 0.07
DOC (mg·g^{-1})	0.020±0.003	0.603 ± 0.04
P extractable (mg·kg^{-1})	84.52±3.01	58.36 ± 4.28
Total PTEs concentration (mg·kg^{-1})		
Total Sb	u.d.l.	u.d.l.
Total As	u.d.l.	u.d.l.
Total Cd	u.d.l.	u.d.l.
Total Fe	524.8±12.7	5494 ± 66
Total Mn	358.1±5.1	147.9 ± 6.2
Total Pb	u.d.l.	u.d.l.
Total Cu	207.1±2.9	u.d.l.
Total Zn	u.d.l.	26.91 ± 0.89
